# Griscelli syndrome type 2: A rare case report of pediatric immunodeficiency and neurological implications

**DOI:** 10.1097/MD.0000000000046420

**Published:** 2026-01-02

**Authors:** Malaka Abubakir, Noura Abdul Rahman, Adel Hamza, Sarah Haj Kasem, Mohammad Ali Khayata, Doaa Kouja

**Affiliations:** a Faculty of Medicine, University of Aleppo, Aleppo, Syrian Arab Republic; b Department of Dermatology, Aleppo University Hospital, University of Aleppo, Aleppo, Syrian Arab Republic.

**Keywords:** albinism, case report, genetic disorder, Griscelli syndrome, immunodeficiency, type 2

## Abstract

**Rationale:**

Griscelli syndrome (GS) is a rare autosomal recessive disorder marked by partial oculocutaneous albinism, immunodeficiency, and neurological issues. It has 3 types based on genetic mutations. This report focuses on a patient with GS type 2, characterized by immune abnormalities and neurological symptoms, with only 160 cases documented globally.

**Patient concerns:**

A 1-year-old boy presented with hyperthermia, diarrhea, and vomiting, revealing hypopigmented skin and silvery-gray hair. He exhibited tachycardia, abdominal distension, hepatosplenomegaly, and signs of immunocompromise. Neurologically, he showed developmental delays and hyperreflexia. Lab tests indicated anemia, thrombocytopenia, and elevated triglycerides.

**Diagnoses:**

Based on clinical history and laboratory tests diagnosed with GS type 2.

**Interventions and outcomes:**

The patient received symptomatic treatment, antibiotics, and frequent transfusions, with strict infection prevention due to limited resources for stem cell transplantation.

**Lessons:**

GS, identified in 1978, is a rare autosomal recessive disorder marked by partial albinism and immunodeficiency, with around 160 cases primarily from the Mediterranean. It comprises 3 types: GS1, GS2, and GS3, each with unique genetic and clinical features. GS1 exhibits partial albinism and neurological issues without immune effects due to MYO5A mutations. GS2 presents severe immunodeficiency and risks such as hemophagocytic lymphohistiocytosis linked to RAB27A mutations. GS3, caused by melanophilin gene mutations, has a better prognosis. Diagnosis involves hair microscopy and genetic testing, and while supportive treatments exist, early diagnosis is vital for improved outcomes. GS is a rare genetic disorder with varied symptoms, categorized into 3 types, requiring genetic testing for diagnosis and treatment ranging from management to stem cell transplantation.

## 1. Introduction

Griscelli syndrome (GS) is a rare autosomal recessive disorder characterized by partial oculocutaneous albinism, immunodeficiency, and neurological manifestations.^[1]^ The first case was reported in 1978.^[1]^ GS is classified into 3 types based on the underlying genetic mutations and clinical features.^[2]^ A hallmark of the syndrome is the presence of silvery-gray hair and hypopigmented skin.^[2]^

GS type 1 is caused by mutations in the MYO5A gene, which plays a critical role in organelle transport within neuronal cells and melanocytes. This leads to partial albinism and neurological deficits.^[1]^ Clinically, patients with GS type 1 exhibit significant neurological alterations, including developmental delays, seizures, intellectual disabilities, and hypotonia (weak muscle tone).^[2]^

GS type 2 results from mutations or dysregulation of the RAB27A gene, which is particularly important in specific cell types such as melanocytes, cytotoxic T lymphocytes, and NK cells.^[1]^ Affected individuals are prone to immune system abnormalities and may develop hemophagocytic syndrome due to the overactivity of macrophages and T lymphocytes.^[2]^

GS type 3 arises from mutations in the MLPH gene, which encodes melanophilin.^[3]^ This type is characterized solely by hypopigmented skin, without associated immunodeficiency or neurological symptoms.^[2,3]^

In this study, we present a case of a 1-year-old male patient diagnosed with GS type 2 without prior genetic examination. This syndrome is extremely rare, with only about 160 reported cases worldwide.^[3]^

This manuscript was prepared in accordance with the CARE 2013 guidelines.^[4]^

## 2. Case presentation

**Figure 1.**
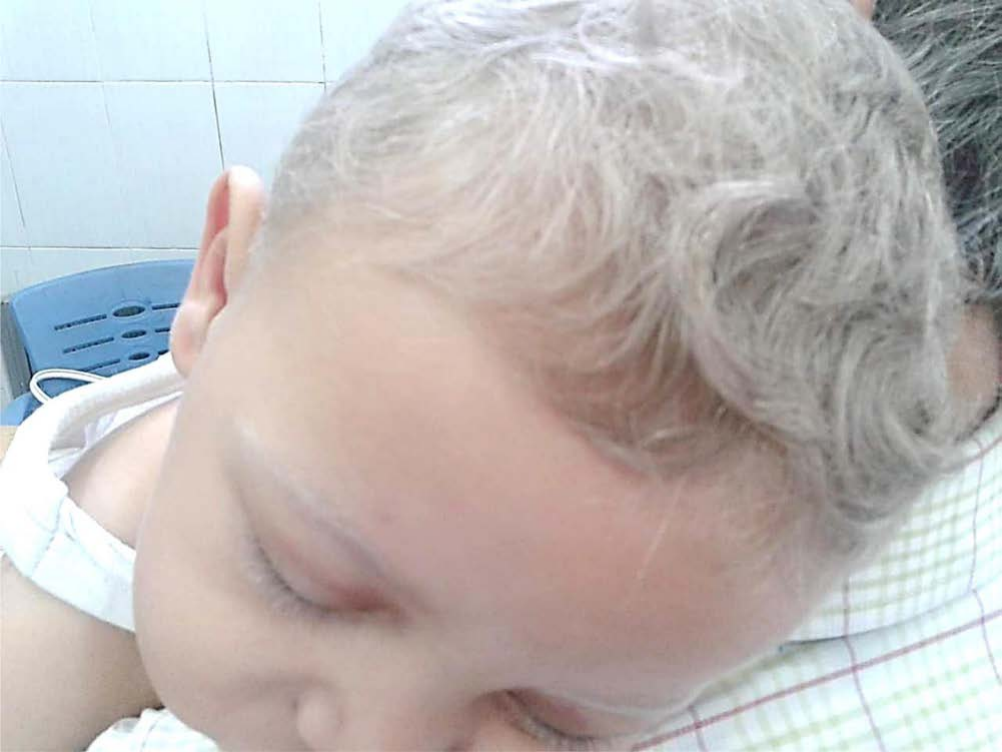
The patient shows dermatologic symptoms of silvery-gray hair on the scalp, eyebrows, and eyelashes.

A 1-year-old male child was brought to the hospital with hyperthermia, diarrhea, and vomiting, in addition to an upper respiratory tract infection. The patient had been diagnosed with a chronic upper respiratory tract infection prior to presentation at our center by another physician. According to the caregivers, he had received antibiotic treatment for this condition; however, the exact antibiotic regimen and duration were not recalled. Upon examination, hypopigmented areas were noted on the arms and legs, prompting consultation with the dermatology department. Clinical inspection revealed silvery-gray hair on the scalp, eyebrows, and eyelashes (Fig. 1), as well as hypopigmentation of the limbs. There were no signs of bleeding or bruising, and the mucous membranes and nails appeared normal.

Physical examination revealed tachypnea, tachycardia, and abdominal distension. Abdominal ultrasound demonstrated hepatosplenomegaly with the following findings: liver longitudinal diameter of 77.5 mm without biliary duct dilatation; contracted gallbladder with edematous wall; spleen longitudinal diameter of 77.2 mm, indicating mild splenomegaly; kidneys bilaterally normal in size and echogenicity; urinary bladder nearly empty; and no free intraperitoneal fluid. These clinical and imaging findings were suggestive of Griscelli syndrome (GS).

The definitive diagnostic tool (genetic testing) was not available due to infrastructure limitations caused by the ongoing conflict in our country. This hindered our ability to identify the specific mutation involved, and thus, the diagnosis was based on clinical features, patient history, and supporting laboratory and imaging results.

Medical history revealed that the patient had a significant record of recurrent bronchiolitis accompanied by hyperthermia; which was diagnosed a few months ago by the family doctor and treated classically with antibiotics and antipyretics that the parents couldn’t remember their name, suggesting an underlying immunocompromised state. His medication history was limited to repeated antibiotic courses for recurrent infections since birth. No known allergies were reported. Lymph nodes were not enlarged, and there was no history of surgical procedures. Notably, the parents were third-degree cousins, although neither had clinical manifestations of GS.

Neurological examination revealed a closed fontanel, reactive pupils, and no signs of nuchal rigidity. Hyperreflexia was observed in the lower limbs. The child showed psychomotor developmental delay: he required assistance for walking and was nonverbal at the time of presentation.

Laboratory evaluation showed hypochromic microcytic anemia, thrombocytopenia, pancytopenia, and hypertriglyceridemia, while blood glucose and serum electrolyte levels remained within normal limits. Due to severe resource limitations in our setting, advanced immunological assays (e.g., lymphocyte subsets, NK-cell function, quantitative immunoglobulins) were not available at the time of diagnosis. Therefore, the immunodeficiency was inferred from the clinical phenotype, including recurrent severe infections, hematologic abnormalities (pancytopenia, thrombocytopenia), hepatosplenomegaly, and characteristic hypopigmentation, consistent with GS type 2.

**Figure 2.**
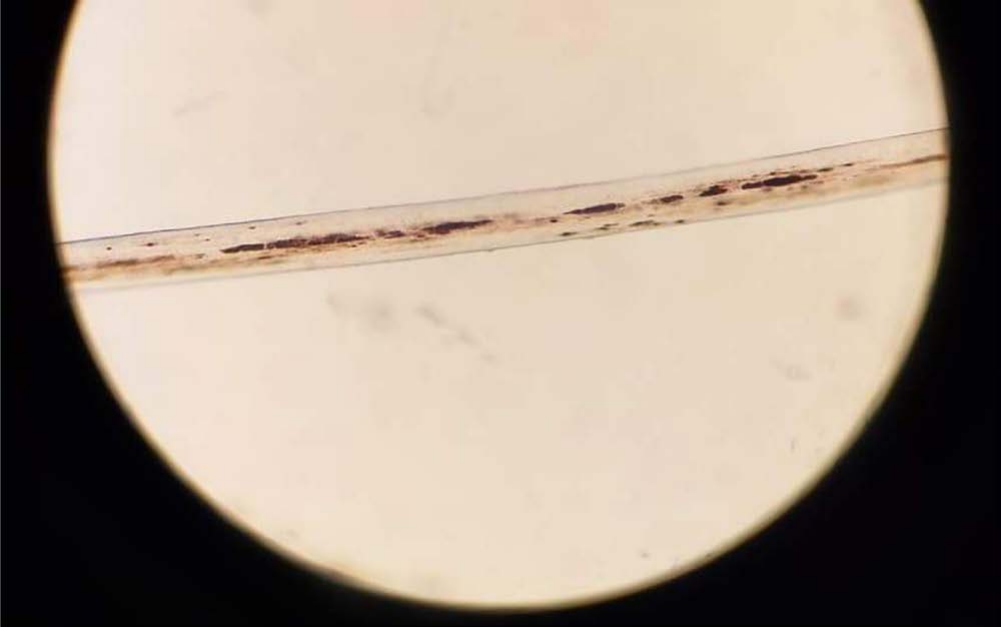
Microscopic examination shows the patient’s hair shaft with irregularly large pigment clusters scattered along the strands.

Microscopic hair shaft analysis, requested by the dermatology team, revealed large, irregularly distributed melanin granules along the strands (Fig. 2), a hallmark feature of GS.

Based on the clinical, neurological, and dermatological findings, a diagnosis of GS type 2 was established. Hematopoietic stem cell transplantation (HSCT) was indicated but could not be performed due to lack of resources in our country. The patient was managed supportively due to the unavailability of bone marrow transplantation in our country. Broad-spectrum antibiotic therapy was initiated with intravenous meropenem (1 g/20 mL) administered at a dose of 6.6 mL 3 times daily, targeting recurrent bacterial infections secondary to immunodeficiency. Regular blood and platelet transfusions were provided on a weekly basis with an approximate volume of 10 to 15 mL/kg per session, targeting correction of anemia to manage anemia and thrombocytopenia. Supportive care included close monitoring of vital signs, liver and spleen size, and neurological status.

Despite the absence of HSCT, which remains the definitive treatment for GS type 2, the patient demonstrated partial clinical improvement with a reduction in infection frequency and stabilization of hematologic parameters.

The patient responded well to treatment with no adverse or unexpected events noted during hospitalization. He was discharged in a stable condition. Follow-up for 2 months showed clinical stability without significant growth or developmental improvement.

Unfortunately, long-term follow-up data and supporting documentation; such as post-discharge ultrasound images were unavailable due to loss of contact with the patient’s family following their displacement during the war. This, combined with the hospital’s limited capacity for data storage, constitutes a key limitation of this case report.

## 3. Discussion

GS was first described in 1978 as a condition characterized by partial albinism associated with immunodeficiency.^[3,5]^ Since then, approximately 160 cases have been documented in the literature, primarily from the Mediterranean region.^[3]^ Current understanding classifies GS as an autosomal recessive genetic disorder, with greater prevalence in countries with higher rates of consanguineous marriage.^[6]^ This rare syndrome is divided into 3 main types, each exhibiting distinct genetic and clinical manifestations while sharing common features, such as partial albinism. This feature can also be seen in other diseases, such as Chédiak-Higashi syndrome (CHS), Hermansky-Pudlak syndrome type 2, and Elejalde syndrome,^[1]^ which makes diagnosing GS particularly challenging.

The differences between these types are significant, and it is crucial to identify the subtype a patient has in order to provide appropriate treatment. Griscelli syndrome type 1 (GS1) is characterized by partial albinism, distinctive gray hair, and prominent neurological impairment, including psychomotor retardation and developmental delay, without apparent effects on the immune system. This subtype was once confused with Elejalde syndrome, which also presents with silvery gray hair and central nervous system (CNS) malfunctions similar to those seen in GS1. However, Elejalde syndrome is now considered a subset of GS type 1.^[5]^ GS1 is caused by genetic mutations affecting the myosin Va (MYO5A) gene located on chromosome 15q21, which is responsible for regulating organelle transport in melanocytes and neuronal cells. This abnormal pattern of melanosome transport alters intracellular transport via vesicles, leading to a significant reduction in fast axonal transport in nerve cells. These combined manifestations contribute to the clinical symptoms of GS1, including alterations in axonal transport, dendritic spine structure, synaptic plasticity, and impaired neuronal development.^[1,5]^ Currently, there is no cure for GS1. Management of the disorder is limited to palliative and supportive care.^[7]^

Another disease that can be confused with Griscelli syndrome is CHS, which presents with albinism, recurrent infections, and a reduced Nitro Blue Tetrazolium test. Skin, hair, and Schwann cell biopsies in CHS display aberrant membrane-bound lysosome-like organelles and aberrant vacuolation in granulocytes in peripheral blood films.^[8]^ These characteristics can mimic the immunodeficiency seen in GS2. The differentiation between CHS and GS2 can be accomplished using light microscopy of the hair shaft, which shows large irregular clumps of pigmentation in GS2, whereas the pigmentation in CHS is more evenly distributed.^[6]^ GS2 results from a mutation in the RAB27A gene, also located on 15q21, where the GS1 mutation occurs. The RAB27A gene encodes a GTPase that plays a primary role in the peripheral transfer of melanosomes to nearby cells, such as keratinocytes, and in granule exocytosis in cytotoxic T lymphocytes.^[5]^ The mutation leads to the characteristic signs of GS2, including partial albinism, severe immunodeficiency, and secondary neural impairment due to CNS infections resulting from the compromised immune system or due to lymphocytic infiltration of the CNS in cases of hemophagocytic lymphohistiocytosis (HLH), a dangerous complication of GS2.^[6]^ HLH is a systemic inflammatory condition resulting from elevated serum levels of cytokines released by cytotoxic T cells and natural killer (NK) cells, which lead to macrophage hyperactivation. This hyperactivation can cause significant systemic abnormalities such as lymphadenopathy, fever, hepatomegaly, splenomegaly, bicytopenia, hypertriglyceridemia, and hyperferritinemia, making GS2 a potentially fatal condition if not managed properly.^[3,6]^ Treatment options to slow the progression of HLH include dexamethasone, cyclosporine, anti-thymocyte globulin, and alemtuzumab,^[6,9]^ while HSCT remains the only potential cure for GS2.^[3]^

Griscelli syndrome type 3 (GS3) represents a restricted expression of the disease, caused by a mutation in the melanophilin gene located at 2q37, which leads to hypopigmentation and the presence of gray hair observed in all GS subtypes.^[5,6]^ GS3 has the best prognosis among the 3 types, as it has no impact on either the nervous or immune systems and requires no form of therapy.^[7]^

As seen in many cases, early diagnosis plays a crucial role in improving the quality of life for patients and increasing the chances of recovery. Therefore, it is essential to have the appropriate diagnostic means available for the early detection of such a rare disorder. Diagnostic methods range from simple approaches, such as hair microscopy and skin biopsies, to more complex procedures like genetic sequencing, which may not always be accessible. Additional tools that can confirm the diagnosis of GS include degranulation assays and assessments of serum immunoglobulin levels, as well as HLH indicators such as NK cell activity, serum ferritin, serum fibrinogen, and bone marrow aspiration cytology.^[3]^ In general, the syndrome has a poor prognosis and is most likely fatal due to HLH complications, alongside respiratory failure and intracranial hemorrhage, despite ongoing treatment efforts.^[6]^

## 4. Conclusion

GS is a rare genetic disorder, primarily resulting from a gene expression disorder and often associated with consanguineous marriages.

The associated signs and symptoms of GS encompass a range of neurological disorders and dermatological manifestations, in addition to immune system complications. However, the clinical presentation varies according to the specific type of the syndrome, which is categorized into 3 types. The most severe form, Griscelli syndrome type 2 (GS2), requires prompt diagnosis and treatment to prevent life-threatening complications, while griscelli syndrome type 1 (GS) 1 is limited with neurological manifestations and hypopigmentation. In contrast, Griscelli syndrome type 3 (GS3) is characterized by hypopigmentation without significant health effects and does not require therapeutic intervention.

Genetic testing is the definitive method for diagnosing GS, as it allows for the identification of the specific mutation involved. Treatment options range from symptomatic management to HSCT, depending on the severity and type of the syndrome.

## Author contributions

**Conceptualization:** Sarah Haj Kasem, Doaa Kouja.

**Data curation:** Malaka Abubakir.

**Project administration:** Malaka Abubakir.

**Writing – original draft:** Malaka Abubakir, Adel Hamza, Sarah Haj Kasem, Mohammad Ali Khayata, Doaa Kouja.

**Writing – review & editing:** Malaka Abubakir, Noura Abdul Rahman.
